# Genetic Diversity and Pathogenicity of *Rhizoctonia* spp. Isolates Associated with Red Cabbage in Samsun (Turkey)

**DOI:** 10.3390/jof7030234

**Published:** 2021-03-21

**Authors:** Ismail Erper, Goksel Ozer, Ruslan Kalendar, Sirin Avci, Elif Yildirim, Mehtap Alkan, Muharrem Turkkan

**Affiliations:** 1 Department of Plant Protection, Faculty of Agriculture, Ondokuz Mayis University, Atakum, 55139 Samsun, Turkey; avci.sirin@hotmail.com (S.A.); elf.yldrm@hotmail.com (E.Y.); 2 Department of Plant Protection, Faculty of Agriculture, Kyrgyz Turkish Manas University, Bishkek 720044, Kyrgyzstan; 3 Department of Plant Protection, Faculty of Agriculture, Bolu Abant Izzet Baysal University, 14030 Bolu, Turkey; gokozer@gmail.com (G.O.); alkanmhtp@gmail.com (M.A.); 4 Department of Agricultural Sciences, University of Helsinki, 00014 Helsinki, Finland; 5 National Laboratory Astana, Nazarbayev University, Nur-Sultan 010000, Kazakhstan; 6 Department of Plant Protection, Faculty of Agriculture, Ordu University, 52200 Ordu, Turkey; muharremturkkan@gmail.com

**Keywords:** red cabbage, *Rhizoctonia* spp., anastomosis groups, iPBS, pathogenicity

## Abstract

A total of 132 *Rhizoctonia* isolates were recovered from red cabbage plants with root rot and wirestem symptoms in the province of Samsun (Turkey) between 2018 and 2019. Based on the sequence analysis of the internal transcribed spacer (ITS) region located between the 18S and 28S ribosomal RNA genes and including nuclear staining, these 124 isolates were assigned to multinucleate *Rhizoctonia solani*, and eight were binucleate *Rhizoctonia*. The most prevalent anastomosis group (AG) was AG 4 (84%), which was subdivided into AG 4 HG-I (81%) and AG 4 HG-III (3%), followed by AG 5 (10%) and AG-A (6%), respectively. The unweighted pair group method phylogenetic tree resulting from the data of 68 isolates with the inter-PBS amplification DNA profiling method based on interspersed retrotransposon element sequences confirmed the differentiation of AGs with a higher resolution. In the greenhouse experiment with representative isolates (*n* = 24) from AGs on red cabbage (cv. Rondale), the disease severity index was between 3.33 and 4.0 for multinucleate AG isolates and ranged from 2.5 to 3.17 for AG-A isolates. In the pathogenicity assay of six red cabbage cultivars, one isolate for each AG was tested using a similar method, and all cultivars were susceptible to AG 4 HG-I and AG 4 HG-III isolates. Redriver and Remale were moderately susceptible, while Rescue, Travero, Integro, and Rondale were susceptible to the AG 5 isolate. The results indicate that the most prevalent and aggressive AGs of *Rhizoctonia* are devastating pathogens to red cabbage, which means that rotation with nonhost-crops for these AGs may be the most effective control strategy. This is the first comprehensive study *of Rhizoctonia* isolates in red cabbage using a molecular approach to assess genetic diversity using iPBS-amplified DNA profiling.

## 1. Introduction

Turkey is the world’s fourth-largest vegetable producer with approximately one million hectares of cultivation area and an annual yield of 24.1 million tons [1]. Brassicas is a family of vegetables, including important and highly diverse crops that are widely grown due to their contribution to the human diet and other health benefits [2,3].

The most common cultivated species of the brassicas are broccoli (*Brassica oleracea* var. *italica*), Brussels sprouts (*B. oleracea* var. *gemmifera*), cauliflower (*B*. *oleracea* var. *botrytis*), Chinese cabbage (*B. pekinensis*), kale (*B. oleracea* var. *acephala*), radish (*Raphanus sativus*), red cabbage (*B. oleracea* var. *capitata* f. *rubra*), and white head cabbage (*B*. *oleracea* var. *capitata* f. *alba*), which are responsible for 4.5% of total vegetable production in Turkey. Red cabbage in Samsun province has about 52.266 da of a total cultivation area and 192.219 tons of annual production, accounting for 57% of the country’s total production [4]. Cruciferous vegetables in the family of Brassicaceae are cultivated throughout the world; however, they suffer from various bacterial, fungal, protozoan, and viral diseases [5]. *Rhizoctonia* spp. is an important and difficult-to-treat group among the myriad of fungal pathogens and associated with damping off, root rot, wirestem, foot rot, and the head rot disease complex of brassicas, which result in economic yield losses [5,6,7,8,9,10,11]. This phytopathogen as a complex group shows significant differences in cultural, molecular, and biochemical properties, and pathogenicity.

*Rhizoctonia* isolates can be classified into the groups of multinucleate *Rhizoctonia* (MNR) binucleate *Rhizoctonia* (BNR), and uninucleate *Rhizoctonia* (UNR), taking into account the average number of nuclei per young vegetative hyphal cell of the isolates [6]. Isolates of *Rhizoctonia* spp. are further divided into numerous anastomosis groups (AGs) on the basis of interactions of isolates with hyphal anastomosis that differed in genotypic and phenotypic properties. Thirteen AGs designated as AG1–AG13 and AG-BI have been assigned within MNR, *Rhizoctonia solani* J.G. Kuhn, whilst 19 AGs of BNR, referred to as AG A–AG W, have been designated based on hyphal fusion [12,13,14,15,16]. Some multinucleate AGs are further clustered into distinct subgroups [12,17].

The internal transcribed spacer (ITS) between the 18S and 28S ribosomal RNA genes, including ITS1, 5.8S rRNA, and ITS2, has been widely employed to evaluate genetic variation and characterize AG groups of *Rhizoctonia* isolates [13]. The resolution of hyphal anastomosis analysis is insufficient to distinguish subgroups within AG 1, AG 2, and AG 4, because anastomosis reaction occurs between isolates of various subgroups in the same group [12,18]. The analysis of the ITS sequences is a critical tool to overcome this bottleneck.

The isolates of MNR (AG 1 (-IB and -IC), AG 2 (-1, -2, -2IIIB, and -2IV), AG 3, AG 4 (HG-I, HG-II, and HG-III), AG 5, AG 9, AG 10), and BNR (AG-A, AG-E, AG-Fb, AG-Fc, and AG-K) have been noted to cause destructive diseases in *Brassica* spp. in Australia [19], Belgium [20], Canada [21], China [7], Japan [9,22,23], Brazil [18], North America [24,25], the United Kingdom [26], Turkey [10,11], and Vietnam [8].

However, the genetic conservationism of the internal transcribed spacer sequences for a particular species makes the study of these sequences for intraspecific diversity unsuitable. Ideal and simple approaches that may correspond to the assessment of genetic intraspecific diversity are based on DNA profiling methods. Such DNA profiling methods include all PCR-based DNA fingerprinting method variants of the Random Amplified Polymorphic DNA (RAPD) method, such as Inter Simple Sequence Repeat (ISSR), and others [27].

Fundamental components of all eukaryote genomes include mobile genetic elements and other interspersed repeats that can activate under stress conditions and indirectly promote survival under environmental stresses. Various PCR-based DNA fingerprinting methods are used to detect chromosomal changes related to recombination processes of mobile genetic elements [28,29,30]. These methods are based on interspersed repeat sequences and are an effective approach to assess the biological diversity of hosts and their variability. The assessment of genetic intraspecific diversity using mobile genetic elements or other interspersed repeats is simple and accessible as RAPD. Most disseminated repeats with one other during inter- and intrachromosomal recombination, which leads to the formation of inverted repeats that are close to one other and allow PCR amplification [27,31]. These interspersed repeat sequences arise from various families of long-terminal repeat (LTR) retrotransposons that have replicated through RNA reverse transcription and integration of resultant cDNA into another locus [32]. Highly conserved repeat sequences for these retrotransposons include the tRNA priming binding site (PBS) when initializing retrotransposon replication. Sequences of the PBS region are complementary to at least 12 nucleotides of the tRNA sequences, which are sufficient for use as PCR primers [33]. As retrotransposon sequences are frequently near one other in inverted orientation, PBS sequences are accessible when used for DNA amplification for most eukaryotic species with large genomes, such as plants and animals [28].

Genetic differences in the AGs and their subgroups were genetically evaluated using DNA profiling PCR methods based on a single-primer complementary to the PBS region downstream of the 5′ LTR for retrotransposons [33]. This DNA marker system based on retrotransposons, the Inter Primer Binding Site (iPBS) amplification technique, is ideal for the assessment of the genetic intraspecific diversity of all eukaryotes [33,34]. This PCR-based DNA fingerprinting method has permitted the genetic differentiation between mold and yeast at intraspecies levels, as well as the identification of the AGs of *Rhizoctonia* spp. [35,36,37,38,39,40]. Genetic differences in hypertension and their subgroups have been phylogenetically assessed using PCR-based DNA fingerprint techniques even in the absence of prior knowledge of the sequences [34,41,42,43,44]. Several studies have reported that *Rhizoctonia* isolates within many AGs have caused diseases in important crops in Turkey to date [11,45,46,47,48,49]; however, there are no detailed reports identifying the AGs and their subgroups of Rhizoctonia isolates causing the root rot and wirestem of red cabbage in Turkey. No information is available about the aggressiveness of the red cabbage isolates to red cabbage cultivars commonly cultivated in the country. The aims of this study were the following: (i) to conduct the molecular characterization of AGs and their subgroups of *Rhizoctonia* isolates recovered from diseased red cabbage plants showing root rot and wirestem symptoms; (ii) to apply DNA-profiling PCR methods based on iPBS amplification to discriminate AGs of *Rhizoctonia* spp.; (iii) to determine the pathogenicity of the isolates belonging to AGs determined in this study in red cabbage cv. Rondale; and (iv) to reveal the resistance status of six red cabbage cultivars against different AGs under greenhouse conditions.

## 2. Materials and Methods

### 2.1. Sample Collection and Isolation of Rhizoctonia Isolates

During the 2018 and 2019 growing seasons, diseased plants with typical symptoms of root rot and wirestem were sampled from 79 randomly selected commercial red cabbage fields in Samsun province between July and October. For each field, three diseased plants were quickly collected onto Whatman filter paper and shipped at room temperature to the laboratory.

Necrotic basal stem and root tissues were washed under tap water, rinsed in sterile distilled water, and cut into 1–2 cm sections. These segments were surface sterilized in 1% NaClO solution for 2 min, blotted-dry on sterile filter papers, and placed on 2% acidified water agar (pH 4.5) with 10% lactic acid. The plates were incubated at 23 °C in the dark for two days. Following two-day incubation, the hyphae of fungal colonies recovered from the sections were examined under a CX31 compound microscope (Olympus, Tokyo, Japan) at a magnification of 200×. Colonies showing *Rhizoctonia*-like growth [50] were subjected to a second transfer to potato dextrose agar (PDA; 213400, BD Difco, Sparks, MD, USA) and incubated at 23 °C for one week in the dark. The isolates were maintained at 4 °C on PDA slants.

To specify the number of nuclei per hyphal cell of *Rhizoctonia* isolates, the 2-day-old hyphal tips of each isolate were stained with 0.5% safranin O and 3% KOH solution as described by Bandoni [51] and observed immediately at 400× by the CX31 microscope.

### 2.2. DNA Extraction

The mycelial discs from the solid culture of isolates were transferred to 250 mL Erlenmeyer flasks each containing 80 mL potato dextrose broth (254920, BD Difco, Sparks, MD, USA) placed on a shaking incubator at 180 rpm and 23 °C for three days. Mycelia were harvested by filtration, washed three-times with sterile distilled water, blotted dry, and ground to a powder in liquid nitrogen. Approximately 50–100 mg of the mycelial powder for each isolate was subjected to genomic DNA extraction with the CTAB method, as described in [52]. The DNA pellets were dissolved in 1 × TE buffer (1 mM EDTA, 10 mM Tris-HCl, pH 8.0). DNA concentration was measured using a nanospectrophotometer (DS-11 FX+, Denovix Inc., Wilmington, DE, USA). The resultant DNA for each isolate was diluted with 1 × TE solution to 10 ng/μL and used as a template for PCR experiments.

### 2.3. ITS Sequencing

To amplify the ITS of genomic rDNA from *Rhizoctonia* isolates, the primer pair ITS1/ITS4 [53] was used. The amplifications were carried out in a 50 μL reaction volume containing 25 μL DreamTaq PCR Master Mix (2X) (Thermo Fischer Scientific, Waltham, MA, USA), 0.4 µM of each primer, and 10 ng template DNA. The PCR amplification was carried out as follows: 3 min at 95 °C; 30 cycles of 30 s at 95 °C, 30 s at 52 °C; 1 min at 72 °C; and a final extension of 5 min at 72 °C. The PCR products were purified and bidirectionally sequenced by the Macrogene Inc. Sequencing Service (Seoul, Korea).

Nucleotide sequence comparisons were conducted using the National Center for Biotechnology Information (NCBI) Basic Local Alignment Search Tool (BLAST) network services with ITS sequences as a query to determine the closest available reference sequences of various AGs in the complete NCBI nucleotide library.

The alignment of the sequences of the isolates derived in this study and representative *Rhizoctonia* isolates retrieved from GenBank (AG 4 HG-I: AB000012, HQ636466, AY387544; AG 4 HG-III: AF354077, JF701709, HQ185372; AG 5: HQ185737, MF070670, KJ170355; AG-A: AY927330, DQ102411, FR734301) was implemented in MEGA X [54] with the CLUSTAL W alignment method [55]. A Maximum Likelihood (ML) tree was constructed in a maximum parsimony (MP) analysis starting tree automatically generated by the software, and the robustness of phylogeny was assessed using 1000 bootstraps [56].

### 2.4. iPBS Amplification Analyses

A total of 7 PBS primers were selected for the assessment of genetic intraspecific diversity based on DNA profiling methods for 68 *Rhizoctonia* isolates belonging to different AGs (Table 1). The iPBS-amplification PCR was carried out in a 25 μL reaction volume containing 12.5 μL DreamTaq PCR Master Mix (2×), 0.04 unit of *Pfu* DNA Polymerase (Thermo Fischer Scientific, Waltham, MA, USA), 0.8 µM of primer, and 10 ng template DNA. The reaction program was set at 95 °C for 3 min, followed by 32 cycles of 30 s at 95 °C, 30 s at 50–62 °C (depending on primers), and 1 min at 72 °C, with a final extension at 72 °C for 5 min. The amplified DNA fragments were analyzed on 1.4% agarose gel using 1 × TAE buffer at 70 V for 5 h and staining with ethidium bromide. PCR products were visualized and imaged under ultraviolet (UV-B) light in the G: BOX F3 gel documentation system (Syngene, Synoptics Ltd., Cambridge, UK). The PCR reaction was conducted twice for each primer to ensure the reproducibility of the results.

A binary matrix was obtained reporting each specific iPBS fragment present (1) or absent (0). The values of polymorphism information content (PIC) and resolving power (RP) were estimated to assess the performance of iPBS markers according to Roldán-Ruiz et al. [57] and Prevost and Wilkinson [58], respectively. Estimates of similarity based on Jaccard’s similarity coefficient were created using the NTSYS-pc version 2.10e program [59]. The similarity matrix was then analyzed using the unweighted pair group method using the arithmetic average (UPGMA) clustering method. The R software package vegan ver. 2.4.4 [60] was used to construct Principal coordinate analyses (PCoA).

The population structure analysis of 68 *Rhizoctonia* isolates with 106 loci was inferred by Bayesian model-based clustering implemented in the program Structure v.2.3.4 [61]. The algorithm was run using a mixed model of 14 independent values (*K*) from 2 to 15 assumed groups, using 100,000 Markov chain Monte Carlo (MCMC) repetitions after a 100,000 burn-in period. The delta *K* (Δ*K*) model and the estimated likelihood values were used to estimate the most probable number of the clusters using STRUCTURE HARVESTER [62].

### 2.5. Pathogenicity Assays

Of 24 isolates, 14 belonged to AG 4 HG-I, 2 to AG 4 HG-III, 4 to AG 5, and 4 to AG-A of *Rhizoctonia* spp., selected on the basis of AGs, and geographical origin was tested for aggressiveness on red cabbage cv. Rondale under greenhouse conditions. The inoculum of each isolate was prepared on wheat seeds using the seed-colonization method of Brayford [63]. The seeds of red cabbage were superficially disinfected in 1% NaOCl solution for 3 min, washed twice with sterilized distilled water, and air-dried on sterile tissue paper in a laminar flow. Each single seed was sown in plastic pots (7 cm in length, 6.5 cm in diameter) filled with a sterilized peat:perlite (2:1, v:v) mixture. The pots were maintained at 20–25 °C with a 16 h photoperiod in a greenhouse.

Six-week-old seedlings at the three true leaf stage were singly transplanted into 10 cm diameter plastic pots (250 mL) containing a sterilized mixture of sandy loam soil:manure:washed sand (2:2:1, v:v:v), and inoculated with 5 g of colonized wheat seeds by placing into the pit dispersedly and surrounded seedling roots at the same time. Controls were inoculated with sterile wheat seeds. Plants were kept in the greenhouse at 20–25 °C. Six replicates were employed for each isolate, with an equal number of control plants. After inoculation, plants were kept in the greenhouse. Twenty-one days after inoculation, plants were gently uprooted, washed with tap water for 5 min, and evaluated for the disease. The disease severity index (DSI) was assessed by evaluating seedling/root symptoms using a slightly modified 1–4 disease severity scale: 1 = no symptom; 2 = basal rot on part of the stem; 3 = basal rot girdled stem superficially, but plants did not wilt; 4 = basal rot deeply girdled stem, stem base was constricted, roots were sometimes detached from stem, and plants wilted [9]. The values of plant height, shoot and root dry weights, and root length were also recorded for each plant. The experimental set-up was a completely randomized design with six red cabbage seedlings per treatment, and the experiment was repeated once. The isolates tested were re-isolated from the symptomatic tissues of the seedlings.

### 2.6. Resistance Response Classification

The reactions of six red cabbage cultivars (Rescue, Travero, Integro, Remale, Redriver, and Rondale) grown commonly in Turkey were estimated using the method described in the pathogenicity experiment against *Rhizoctonia* spp. The isolates of Rs-RC-20 (AG 4 HG-I), Rs-RC-109 (AG 4 HG-III), Rs-RC-118 (AG 5), and R-RC-131 (AG-A) representing each AG were selected for this experiment. The cultivars were grouped according to disease severity on stems and roots. The cultivars were considered resistant (R) if the mean disease severity was ≤1.50, moderately resistant (MR) if the mean disease severity ranged between 1.51 and 2.25, moderately susceptible (MS) if the mean disease severity ranged between 2.26 and 3.00, and susceptible (S) if the mean disease severity was ≥3.01.

### 2.7. Statistical Analysis

All data obtained from pathogenicity tests was subjected to Box-Cox transformation before statistical analyses, since they failed to meet the assumptions of normal distribution (Shapiro–Wilk test) and variance homogeneity (Levene’s test). Each plant growth parameter was separated individually at a probability level of *p* < 0.05 by Fisher’s least significant difference (LSD) test based on the transformed data. On the other hand, due to the nonnormal distribution of disease severity data, analysis was performed with the Dunn-test based on the rank sums using the Kruskal–Wallis pairwise multiple comparisons. To assess the effect of the isolate, cultivar, and isolate–cultivar on disease severity, a nonparametric test of two-way ANOVA, the Scheirer–Ray–Hare (SRH) test, was performed using R software. All other analyses were conducted using the XLSTAT program version 18.07 (Addinsoft Company, New York, NY, USA).

## 3. Results

### 3.1. Rhizoctonia Species and AGs on Red Cabbage

Altogether, 132 isolates of *Rhizoctonia* spp. associated with dark lesions of underground parts of red cabbage plants were obtained from the fields planted in sixteen locations of Samsun province. Using the nuclear staining method, 124 of 132 isolates were classified as MNR, while the remaining isolates (*n* = 8) were BNR (Table 2).

**Figure 1:**
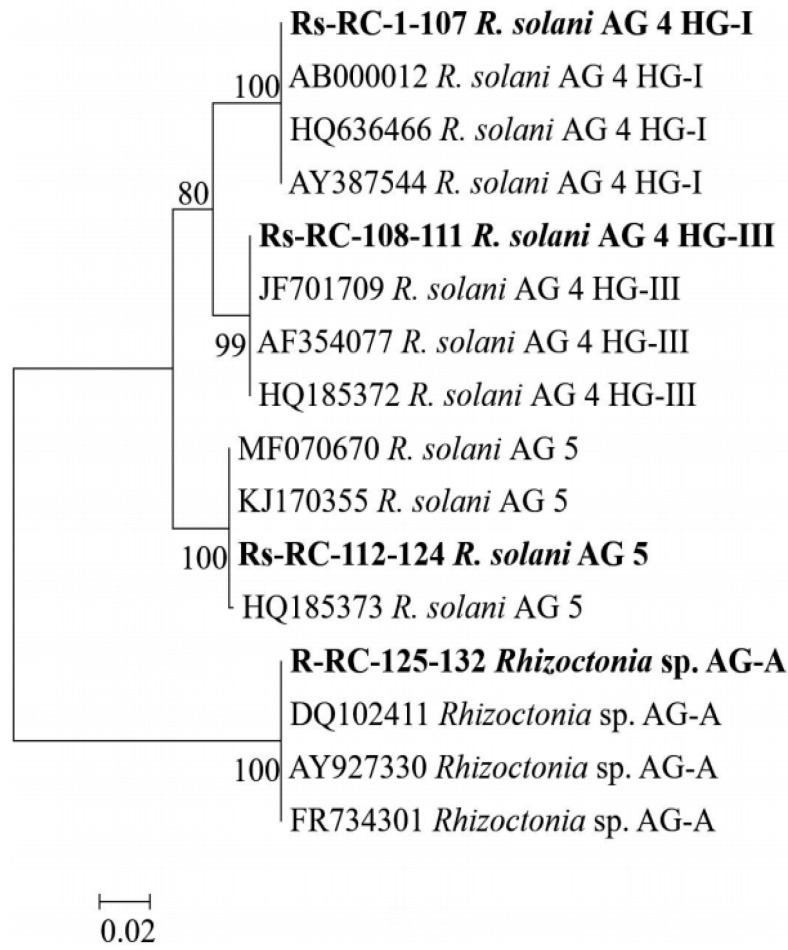
The Maximum Likelihood tree was generated using the ITS sequences of *Rhizoctonia* isolates from this study (bold) and reference isolates representing AGs from GenBank. The percentage of replicate trees in which the associated taxa clustered together in the bootstrap test (1000 replicates) is shown next to the branches.

The BLAST service at the NCBI website was used for analysis of ITS sequences. The BLAST analysis revealed that 107 isolates were MNR AG 4 HG-I, the most prevalent group (81%) in the investigated area, followed by AG 5, AG 4 HG-III, and BNR AG-A with 13 (10%), 4 (3%), and 8 (6%) isolates, respectively. No polymorphism was observed among the nucleotide sequences of isolates within the same AG. The ITS fragments amplified using primers ITS1 and ITS4 showed 100% similarity with those of the corresponding isolates belonging to different AGs from GenBank. The accession numbers provided by GenBank for the sequences obtained in this study are shown in Table 2. The Maximum Likelihood tree generated with the ITS sequences showed a clear clustering among anastomosis groups (Figure 1). All isolates obtained in this study and the known AGs retrieved from GenBank clustered into two major groups with strong bootstrap values (99–100%) according to their nuclei number within vegetative cells. Group I comprised multinucleate isolates, which were further grouped into three subgroups according to their anastomosis groups, whereas the isolates of AG-A formed Group II as the most diverse group.

### 3.2. iPBS Amplification Analysis

**Figure 2:**
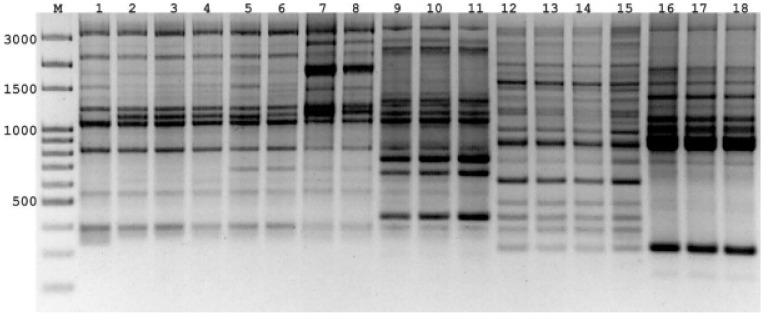
The band profiles with PBS primer (2395) for *Rhizoctonia* isolates. 1–8-AG4 HG-I; 9–11-AG4 HG-III; 12–15–AG5; 16–18-AG-A. M: 100 bp DNA Ladder (Solis BioDyne, Tartu, Estonia).

**Figure 3:**
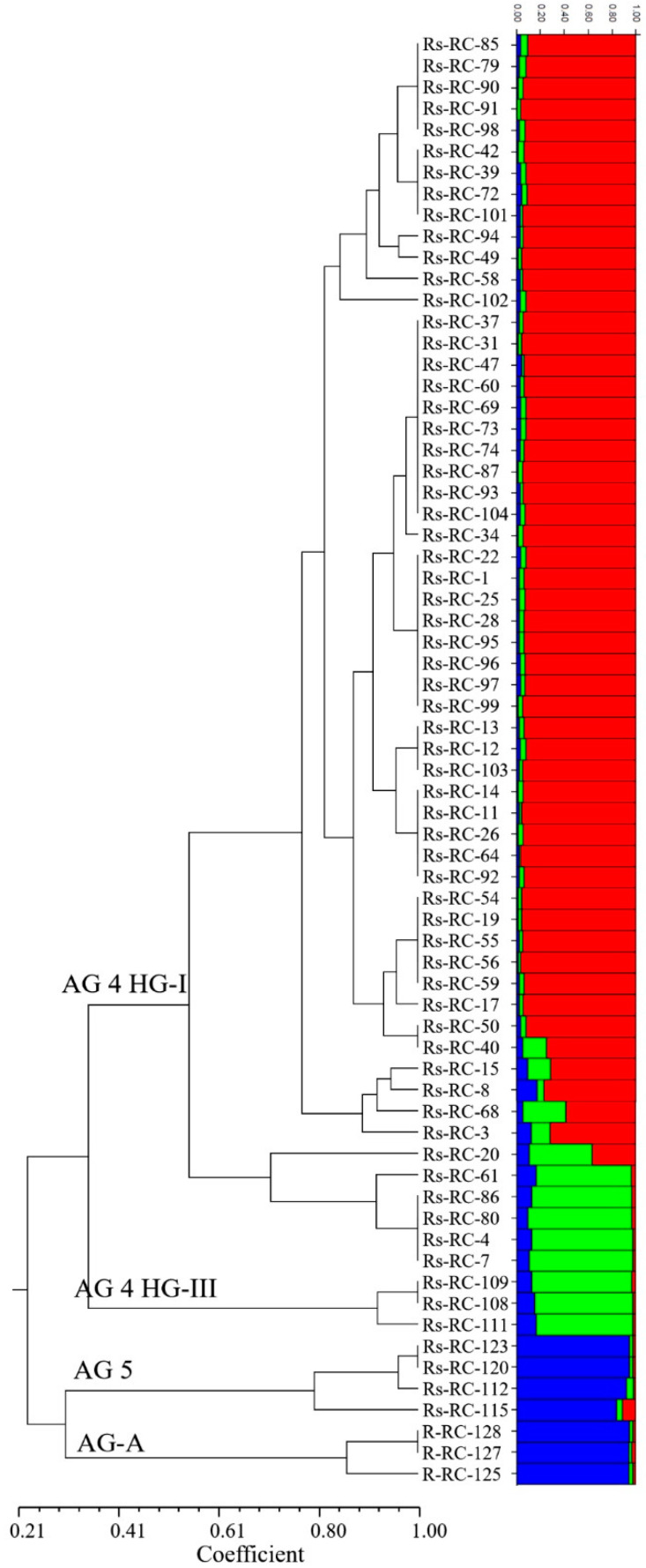
UPGMA tree based on iPBS combined data matrix for 68 *Rhizoctonia* isolates. Each individual is represented by a horizontal bar broken into different colored genetic clusters, with length proportional to the probability of assignment to each cluster.

**Figure 4:**
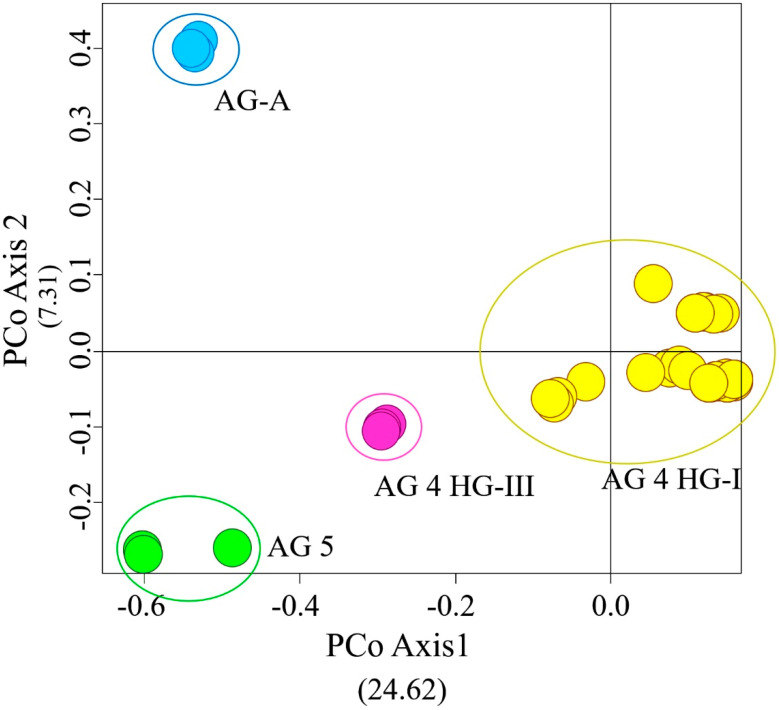
Association matrix for anastomosis groups, based on principal coordinate analyses.

**Figure 5:**
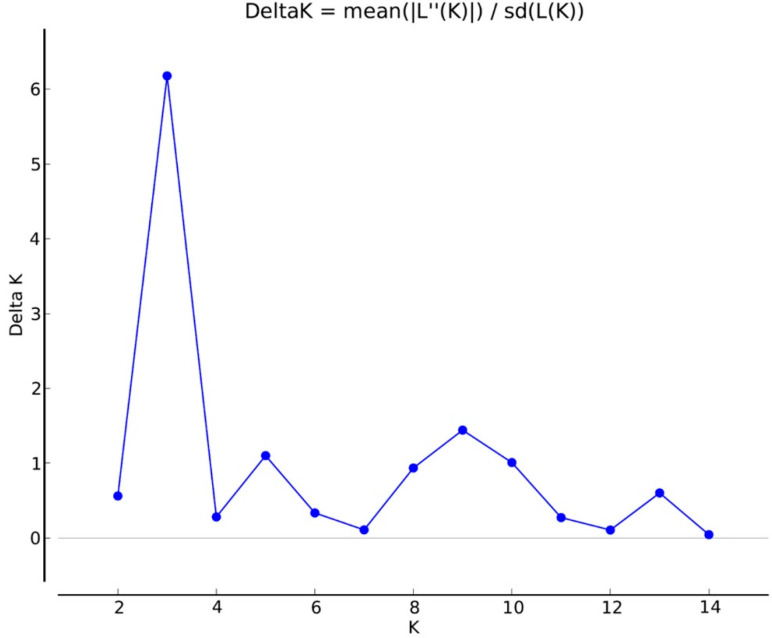
Plot of delta *K* values from the structure analyses of 68 *Rhizoctonia* isolates.

The seven PBS primers in iPBS-amplification profiling generated 106 scorable and reproducible fragments to evaluate the extent of genetic variation between *Rhizoctonia* isolates. Ninety-six of those fragments (90%) were polymorphic. The number of fragments amplified with the primers varied between 11 (2219/2237) and 30 (2395), with a mean of 15 fragments per PBS primer. The values of the PIC and RP index estimated for the markers are listed in Table 1. The range of PIC was 0.37 (2219)–0.14 (2239), averaging 0.18 (Figure 2). The mean of the RP values, a parameter that indicates the discriminatory potential of the primers chosen, was 3.15 for all primers. The highest RP value of 5.32 was obtained from 2395, while the lowest RP value was recorded as 1.94 for 2239. The UPGMA dendrogram produced using the Jaccard’s similarity coefficient for iPBS profiling methods clustered distinctly 68 *Rhizoctonia* isolates into four major groups, which were completely conserved among isolates within the same AG assignment (Figure 3). Group I comprised 58 AG 4 HG-I isolates, and Group 2, 3, and 4 were composed of AG 4 HG-III (3), AG 5 (4), and AG-A (3) isolates, respectively. The groups were further clustered into subgroups at different similarity degrees. In addition, UPGMA cluster analysis based on iPBS-amplification profiling data supported the grouping of isolates based on ITS sequences. The PcoA analysis distinguished *Rhizoctonia* isolates according to their anastomosis groups and plotted the isolates to four major groups, which confirmed the UPGMA pattern (Figure 4). Structure runs were conducted for *K* = 2 to 15 based on the iPBS data. Ln values increased sharply at *K* = 3, after which the increase was slow without reaching the plateau (Figure 5). The highest peak was detected at *K* = 3, which implies that the isolates could be distributed into three separated clusters according to the Δ*K* criteria of Evanno et al. [64].

### 3.3. Virulence of the Isolates

**Figure 6:**
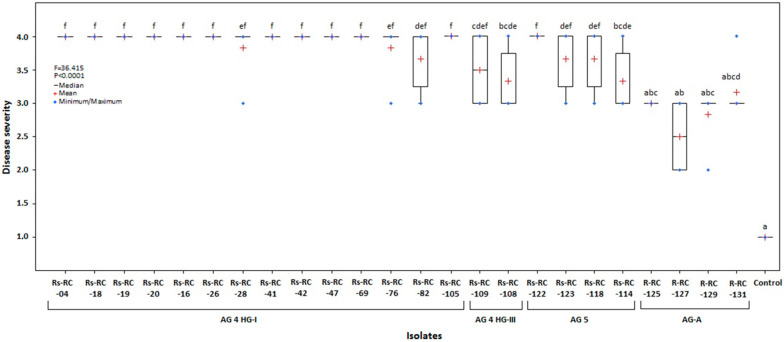
Pathogenicity of *Rhizoctonia* spp. isolates obtained from red cabbage to seedlings of the cv. Rondale twenty-one days after inoculation. Seedling/root symptoms were evaluated 21 days after inoculation based on the following scale: 1 = no symptom; 4 = basal rot deeply girdled stem, stem base was constricted, roots were sometimes detached from stem, and plants wilted. Values followed by the same letter are not significantly different according to Dunn-test based on the rank sums using the Kruskal–Wallis (*p* < 0.0001).

**Figure 7:**
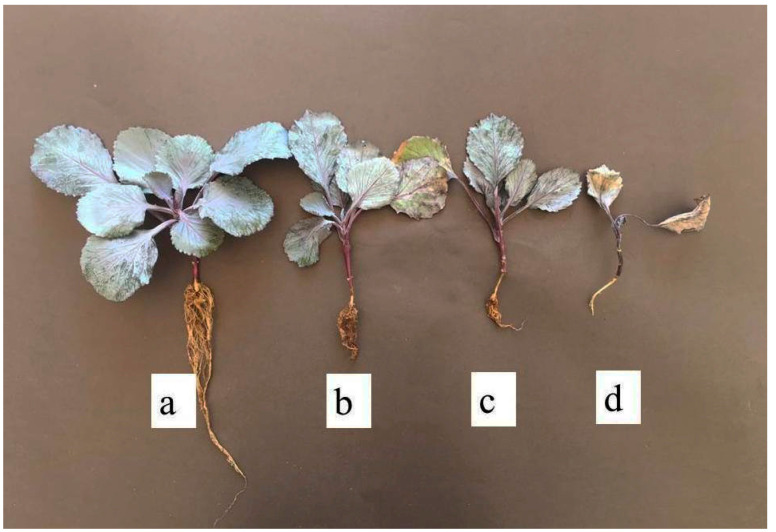
Different disease symptoms caused by isolates belonged to different AGs of *Rhizoctonia* spp. on cv. Rondale seedlings after three-weeks of inoculation: a = healthy seedling (control); b = very little superficial lesions on part of stem (R-RC-127); c = basal rot girdled stem superficially, but plants did not dead (R-RC-125); d = severe basal rot deeply girdled stem, typical stem rot (wirestem), partially restricted root length, and dead plant (Rs-RC-20).

The results of the pathogenicity test on seedlings of red cabbage cv. Rondale revealed a significant difference in virulence between the isolates tested (Kruskal–Wallis ANOVA, *H_24_*_,*125*_ = 106.81, *p* < 0.0001) (Figure 6). Disease symptoms developed in inoculated red cabbage seedlings were scored after twenty-one days of inoculation (Figure 7). The disease severity index (DSI) varied from 3.33 to 4.0 for all *R*. *solani* isolates. In particular, almost all AG 4 HG-I isolates, and isolate Rs-RC-122 of AG 5, caused severe necrosis symptoms in the seedlings, resulting in damping off. All AG-A isolates had a lower virulence ranging from 2.5 to 3.17. All isolates caused a significant reduction in the dry weight of shoots and roots compared to control plants (ANOVA, *F_24_*_,*125*_ = 7.002, *p* < 0.0001; *F_24_*_,*125*_ = 3.369, *p* < 0.0001, respectively) (Table 3). Compared to the control, some *R*. *solani* isolates and all AG-A isolates were found to have no adverse effects on root length or/and plant height (ANOVA *F_24_*_,*125*_ = 2.554, *p* < 0.0004; *F_24_*_,*125*_ = 4.051, *p* < 0.0001, respectively).

### 3.4. Host Response of Some Commercial Cultivars to Different AGs of Rhizoctonia

A statistically significant difference was observed among different AGs (AG 4 HG-I, -HG-III, and 5) of *Rhizoctonia* isolates in the severity of root rot in the cultivars (Rescue, Travero, Integro, Remale, Redriver, and Rondale) of red cabbage (SRH test: isolates: *H_4_* = 135.85, *p* < 0.0001; cultivars: *H_5_* = 11.45, *p* < 0.0432, respectively) (Table 4). In general, the AG-A isolate caused low disease severity in all cultivars, but *R*. *solani* isolates led to the development of severe root rot resulting in high disease severity values ranging from 2.5 to 4. All cultivars were susceptible to AG 4 HG-I and HG-III isolates of *R*. *solani*. Redriver and Remale to the AG 5 isolate were moderately susceptible, while Rescue, Travero, Integro, and Rondale were susceptible. On the other hand, Redriver was moderately resistant to AG-A; Remale, Rescue, and Travero were moderately susceptible; and Integro and Rondale were susceptible. No significant isolate–cultivar interaction was observed for root rot severity on red cabbages (SRH test: isolates:cultivars *H_20_* = 15.09, *p* < 0.7712).

## 4. Discussion

This is the first characterization of *Rhizoctonia* isolates obtained from red cabbage growing areas in Turkey. To the best of our knowledge, no reports about the pathogenicity of *Rhizoctonia* spp. in red cabbage plants have been recorded in the world. The majority of the *Rhizoctonia* isolates were MNR AG 4 (84%). The sequence analysis of the ITS allowed further division of the 111 isolates belonging to MNR AG 4 into two subgroups, AG 4 HG-I (*n* = 107) and AG 4 HG-III (*n* = 4). The remaining isolates were classified into AG 5 (8 isolates) and BNR AG-A (13 isolates).

As indicated in the previous reports, both BNR and MNR isolates cause disease in *Brassica* crops, including white cabbage, kale, broccoli, cauliflower, oilseed rape, and canola in several countries in the world [7,8,9,10,11,18,20,22,23,26,65]. The most prevalent and damaging *R. solani* AGs were AG 2-1 and AG 4 among AGs determined for brassicas [19,24,25,66,67]. Recently, Türkkan, Kılıçoğlu and Erper [11] determined that 37% (11 isolates) of limited *Rhizoctonia* isolates (30 isolates) obtained from kale growing areas in Ordu province, Black Sea region of Turkey, belong to AG 2-1, followed by AG-A with 20% (9 isolates), and AG 4 HG-I with 10% (3 isolates). They isolated *Rhizoctonia* isolates during November–April when temperatures ranged from 7–16 °C. However, in the present study, surveys were conducted between July and October when the weather was warmer (25–33 °C). Previous studies have shown that the abundance of each AG may depend on the climate. For example, it has been reported in several studies that AG 4 HG-I is dominant in warmer periods, while AG 2-1, AG 1-IB, and AG-BI are dominant during colder periods [68,69,70]. Moreover, Yitbarek et al. [71] observed that AG 4 caused severe root rot in *B. napus* seedlings at temperatures varying between 26 and 35 °C, whereas the pathogenic activity of AG 2-1 was considerably reduced at these temperatures. The relationship between AG and temperature is probably the same for isolates in this study and may explain why we commonly isolate *R. solani* AG 4 HG-I during warmer periods. In addition, previous studies have reported that it is the most common pathogen on cucumber, winter squash, bean, and soybean in the Samsun province of Turkey, where brassicas (cauliflower, white cabbage, and red cabbage) are used as an effective rotational crop [47,72,73]. Given that the pathogen is polyphagous, these alternative hosts likely contributed to the prevalence of *Rhizoctonia* root rot in the fields, which is consistent with Keinath’s findings [74]. Cubeta and Vilgalys [75], and Sharon et al. [76] noted that DNA fingerprinting techniques for studying the genetic variation of *Rhizoctonia* are most suitable at the individual level rather than for the determination of AGs or subgroups within an AG. There is a lack of evidence, however, to support this hypothesis, since no DNA marker system has been tested in grouping a large number of *Rhizoctonia* isolates of different AGs. In the present study, the retrotransposon-based iPBS amplification DNA profiling method differentiated the isolates according to their anastomosis groups, including AG 4 HG-I, HG-III, AG 5, and AG-A, via phylogenetic analysis. A more detailed sub-grouping was observed among the isolates within each AG group formed in the phylogenetic tree, especially among AG 4 HG-I isolates. This is in accordance with other researchers who have grouped *Rhizoctonia* spp. isolates using these markers [35,36]. Pourmahdi and Taheri [35] showed the efficacy of this molecular marker system to determine the AGs of *R. solani* isolates from tomato as a novel universal molecular marker system. iPBS-amplification markers have also been successfully employed for the characterization of several other fungi and yeast [36,37,38,39,40,77,78]. To the best of our knowledge, this study is the first characterization of various *Rhizoctonia* spp. isolates belonging to different AGs obtained from red cabbage using the retrotransposon marker system for DNA fingerprinting.

The pathogenicity studies on red cabbage seedlings showed that the disease severity index (DSI) used in the evaluation of the aggressiveness of *Rhizoctonia* isolates varied widely between 2.5 and 4.0. In the MNR isolates, AG 4 HG-I isolates had the highest index value (DSI: 4.0) on the seedlings, with exceptions of the RS-RC-28, -76, and -82 isolates, and Rs-RC-122 isolate of AG 5. The AG 4 HG-I isolates caused wirestem symptoms by severe necrosis of the stem and root tissues. AG 4 HG-III and AG 5 isolates were more aggressive than isolates of BNR AG-A. Pannecoucque, Van Beneden, and Höfte [20] found that cauliflower plants were more susceptible to AG 1-IC, AG 2–1, AG 2–1 subset Nt, AG 2–2, and AG 4 HG-II isolates than AG 1-IB and AG 5 isolates. None of the AG-A isolates caused symptoms on Chinese and white cabbage, but AG-Fc isolates caused severe lesions [8]. Türkkan, Kılıçoğlu, and Erper [11] noted that except for several isolates within AG 2-1, AG 2-1 and AG 4 HG-I caused severe symptoms and deaths on seedlings of kale cv. Arzuman, but the isolates in AG 5, AG-E, AG-Fb, and AG-K were of relatively low and moderate virulence.

The results of pathogen–cultivar interactions in the current study are consistent with those of Keinath and Farnham [24], who found no cultivar by isolate interaction when testing cultivars for root rot resistance against AG 4 and AG 2-1 of *R. solani*. They also determined that AG 4 caused severe wirestem rot in the seedlings of 12 brassica cultivars (3 each of broccoli, cauliflower, cabbage, and collard) in all experiments, while AG 2-1 caused less root rot in the growth chamber but no root rot in the field. Chinese cabbage and white cabbage seedlings were highly susceptible to *R. solani* AG 4 HG-I in both in vitro and in vivo bioassays conducted by Hua, Bertier, Soltaninejad, and Hofte [8]. Türkkan et al. [11] reported that *R. solani* AG 2-1 and AG 4 HG-I isolates generally caused more severe root rot in the seedlings of kale cv. Arzuman. The present study revealed that the Redriver cultivar was moderately resistant to *Rhizoctonia* AG-A, although no cultivars tested were resistant to AGs of *R. solani*.

In conclusion, the *Rhizoctonia* spp. isolates, whether MNR or BNR, cause devastating diseases in many crops, resulting in economic crop losses. The AG classification of *Rhizoctonia* isolates is a critical approach to characterize the various groups that cause plant diseases. The iPBS-amplification DNA profiling analysis is a simple, effective, and powerful tool to identify AG subgroups. Further studies with other isolates belonging to more AGs are needed to comprehensively investigate the association between grouping within *Rhizoctonia* spp. isolates and iPBS-amplification DNA profiling.

In this study, we confirmed the presence of some virulent *Rhizoctonia* isolates associated with root rot and wirestem in red cabbage. Due to the persistence of *Rhizoctonia* in the soil for several years and the presence of different AGs that are aggressive in various host plants [6], crop rotational strategies alone are insufficient to control *Rhizoctonia* in the fields surveyed. The isolates of AG 4 HG-I cause problems for several crops cultivated in the agricultural areas of the Black Sea region, including the province of Samsun [10,11]. AG 4 HG-I, therefore, has the potential to damage Brassica crops. Although the use of chemical fungicides is environmentally undesirable, the most common method for protecting crops against *Rhizoctonia* isolates is the use of fungicides worldwide [79]. In Turkey, several fungicides are used to control *Rhizoctonia* species on different crops as seed or seedling treatments. An approach of integrated management of plant disease including applying chemical and biological fungicides, crop rotation, and breeding varieties with resistance is needed for the efficient control of the root rot and wirestem of red cabbage.

## Figures and Tables

**Table 1:** The information of PBS primers used to evaluate *Rhizoctonia* spp. isolates.

ID	Primer Sequences (5′–3′)	Ta (°C)	GC (%)	TB	PB	PIC	RP
2078	GCGGAGTCGCCA	62.8	75.0	17	15	0.16	3.26
2095	GCTCGGATACCA	53.7	58.3	13	12	0.18	2.82
2219	GAACTTATGCCGATACCA	53.0	44.4	11	9	0.24	3.41
2222	ACTTGGATGCCGATACCA	53.0	50.0	12	10	0.22	3.26
2237	CCCCTACCTGGCGTGCCA	55.0	72.2	11	10	0.16	2.00
2239	ACCTAGGCTCGGATGCCA	55.0	61.1	12	11	0.14	1.94
2395	TCCCCAGCGGAGTCGCCA	52.8	72.2	30	29	0.15	5.32
	Total			106	96		
	Avg.			15.14	13.71	0.18	3.15

Ta (°C), annealing temperature; TB, total band; PB, polymorphic band; PIC, polymorphism information content; RP, resolving power.

**Table 2:** Anastomosis groups, isolate numbers, and location of isolates of *Rhizoctonia* spp. including their accession numbers.

Anastomosis Group	Isolate Denomination *	Location	GenBank Accession Nos.
AG 4 HG-I	Rs-RC-1	Ağıllar	MT068296
	Rs-RC-2	Ağıllar	MT068297
	Rs-RC-3	Ağıllar	MT068298
	Rs-RC-4	Ağıllar	MT068299
	Rs-RC-5	Ağıllar	MT068300
	Rs-RC-6	Ağıllar	MT068301
	Rs-RC-7	Altınova	MT068302
	Rs-RC-8	Altınova	MT068303
	Rs-RC-9	Altınova	MT068304
	Rs-RC-10	Altınova	MT068305
	Rs-RC-11	Altınova	MT068306
	Rs-RC-12	Altınova	MT068307
	Rs-RC-13	Altınova	MT068308
	Rs-RC-14	Altınova	MT068309
	Rs-RC-15	Balıklar	MT068310
	Rs-RC-16	Balıklar	MT068311
	Rs-RC-17	Çetinkaya	MT068312
	Rs-RC-18	Çetinkaya	MT068313
	Rs-RC-19	Dedeli	MT068314
	Rs-RC-20	Dereköy	MT068315
	Rs-RC-21	Dereköy	MT068316
	Rs-RC-22	Doğanca	MT068317
	Rs-RC-23	Doğanca	MT068318
	Rs-RC-24	Doğanca	MT068319
	Rs-RC-25	Doğanca	MT068320
	Rs-RC-26	Karpuzlu	MT068321
	Rs-RC-27	Karpuzlu	MT068322
	Rs-RC-28	Karpuzlu	MT068323
	Rs-RC-29	Karpuzlu	MT068324
	Rs-RC-30	Karpuzlu	MT068325
	Rs-RC-31	Karpuzlu	MT068326
	Rs-RC-32	Karpuzlu	MT068327
	Rs-RC-33	Karpuzlu	MT068328
	Rs-RC-34	Karpuzlu	MT068329
	Rs-RC-35	Karpuzlu	MT068330
	Rs-RC-36	Karpuzlu	MT068331
	Rs-RC-37	Karpuzlu	MT068332
	Rs-RC-38	Karpuzlu	MT068333
	Rs-RC-39	Karpuzlu	MT068334
	Rs-RC-40	Karpuzlu	MT068335
	Rs-RC-41	Karpuzlu	MT068336
	Rs-RC-42	Kaygusuz	MT068337
	Rs-RC-43	Kaygusuz	MT068338
	Rs-RC-44	Kaygusuz	MT068339
	Rs-RC-45	Kaygusuz	MT068340
	Rs-RC-46	Kaygusuz	MT068341
	Rs-RC-47	Kuşçular	MT068342
	Rs-RC-48	Kuşçular	MT068343
	Rs-RC-49	Osmanbeyli	MT068344
	Rs-RC-50	Osmanbeyli	MT068345
	Rs-RC-51	Osmanbeyli	MT068346
	Rs-RC-52	Osmanbeyli	MT068347
	Rs-RC-53	Osmanbeyli	MT068348
	Rs-RC-54	Osmanbeyli	MT068349
	Rs-RC-55	Osmanbeyli	MT068350
	Rs-RC-56	Osmanbeyli	MT068351
	Rs-RC-57	Üçpınar	MT068352
	Rs-RC-58	Osmanbeyli	MT068353
	Rs-RC-59	Osmanbeyli	MT068354
	Rs-RC-60	Osmanbeyli	MT068355
	Rs-RC-61	Osmanbeyli	MT068356
	Rs-RC-62	Osmanbeyli	MT068357
	Rs-RC-63	Osmanbeyli	MT068358
	Rs-RC-64	Osmanbeyli	MT068359
	Rs-RC-65	Osmanbeyli	MT068360
	Rs-RC-66	Osmanbeyli	MT068361
	Rs-RC-67	Osmanbeyli	MT068362
	Rs-RC-68	Şeyhören	MT068363
	Rs-RC-69	Şeyhören	MT068364
	Rs-RC-70	Şeyhören	MT068365
	Rs-RC-71	Şeyhören	MT068366
	Rs-RC-72	Türbe	MT068367
	Rs-RC-73	Türbe	MT068368
	Rs-RC-74	Türbe	MT068369
	Rs-RC-75	Türbe	MT068370
	Rs-RC-76	Türbe	MT068371
	Rs-RC-77	Türbe	MT068372
	Rs-RC-78	Türbe	MT068373
	Rs-RC-79	Türbe	MT068374
	Rs-RC-80	Türbe	MT068375
	Rs-RC-81	Türbe	MT068376
	Rs-RC-82	Türbe	MT068377
	Rs-RC-83	Türbe	MT068378
	Rs-RC-84	Türbe	MT068379
	Rs-RC-85	Türbe	MT068380
	Rs-RC-86	Türbe	MT068381
	Rs-RC-87	Türbe	MT068382
	Rs-RC-88	Türbe	MT068383
	Rs-RC-89	Türbe	MT068384
	Rs-RC-90	Üçpınar	MT068385
	Rs-RC-91	Üçpınar	MT068386
	Rs-RC-92	Üçpınar	MT068387
	Rs-RC-93	Üçpınar	MT068388
	Rs-RC-94	Üçpınar	MT068389
	Rs-RC-95	Üçpınar	MT068390
	Rs-RC-96	Üçpınar	MT068391
	Rs-RC-97	Üçpınar	MT068392
	Rs-RC-98	Üçpınar	MT068393
	Rs-RC-99	Üçpınar	MT068394
	Rs-RC-100	Üçpınar	MT068395
	Rs-RC-101	Yeşilyazı	MT068396
	Rs-RC-102	Yeşilyazı	MT068397
	Rs-RC-103	Yeşilyazı	MT068398
	Rs-RC-104	Yeşilyazı	MT068399
	Rs-RC-105	Yeşilyazı	MT068400
	Rs-RC-106	Yeşilyazı	MT068401
	Rs-RC-107	Yeşilyazı	MT068402
AG 4 HG-III	Rs-RC-108	Koşuköy	MT068403
	Rs-RC-109	Türbe	MT068404
	Rs-RC-110	Türbe	MT068405
	Rs-RC-111	Altınova	MT068406
AG 5	Rs-RC-112	Koşuköy	MT068407
	Rs-RC-113	Koşuköy	MT068408
	Rs-RC-114	Koşuköy	MT068409
	Rs-RC-115	Balıklar	MT068410
	Rs-RC-116	Balıklar	MT068411
	Rs-RC-117	Balıklar	MT068412
	Rs-RC-118	Balıklar	MT068413
	Rs-RC-119	Balıklar	MT068414
	Rs-RC-120	Ağıllar	MT068415
	Rs-RC-121	Ağıllar	MT068416
	Rs-RC-122	Ağıllar	MT068417
	Rs-RC-123	Ağıllar	MT068418
	Rs-RC-124	Ağıllar	MT068419
AG-A	R-RC-125	Balıklar	MT053135
	R-RC-126	Koşuköy	MT053136
	R-RC-127	Koşuköy	MT053137
	R-RC-128	Türbe	MT053138
	R-RC-129	Türbe	MT053139
	R-RC-130	Türbe	MT053140
	R-RC-131	Türbe	MT053141
	R-RC-132	Türbe	MT053142

* R, *Rhizoctonia* sp.; Rs, *Rhizoctonia solani*.

**Table 3:** The effect of isolates of *Rhizoctonia* spp. obtained from red cabbage to seedlings of the cv. Rondale twenty-one days after inoculation.

Anastomosis Group	Isolate ^a^	Root		Shoot Dry Weight (g)	Plant Height (cm)
		Dry Weight (g)	Length (cm)		
AG 4 HG-I	Rs-RC-04	0.013 ^b^ ± 0.002 ^c^ cde ^d^	2.083 ± 0.523 h	0.127 ± 0.014 h	4.883 ± 0.190 ijk
	Rs-RC-16	0.014 ± 0.004 cde	3.083 ± 0.688 c–h	0.200 ± 0.016 def	4.700 ± 0.191 k
	Rs-RC-18	0.010 ± 0.005 e	2.000 ± 0.129 h	0.148 ± 0.032 gh	4.833 ± 0.211 jk
	Rs-RC-19	0.013 ± 0.007 cde	2.333 ± 0.247 gh	0.182 ± 0.020 e-h	5.000 ± 0.289 h–k
	Rs-RC-20	0.017 ± 0.007 cde	2.583 ± 0.300 e–h	0.185 ± 0.020 efg	5.333 ± 0.167 e–k
	Rs-RC-26	0.020 ± 0.012 cde	2.717 ± 0.415 d–h	0.232 ± 0.023 b–e	5.167 ± 0.333 f–k
	Rs-RC-28	0.021 ± 0.005 cde	3.167 ± 0.105 b–h	0.212 ± 0.017 def	5.250 ± 0.214 f–k
	Rs-RC-41	0.019 ± 0.005 cde	3.367 ± 0.851 b–h	0.202 ± 0.016 def	5.083 ± 0.539 g–k
	Rs-RC-42	0.019 ± 0.007 cde	3.500 ± 0.129 b-g	0.195 ± 0.023 d–g	5.667 ± 0.279 c–i
	Rs-RC-47	0.013 ± 0.004 cde	3.083 ± 0.455 b–h	0.243 ± 0.010 b–e	5.833 ± 0.307 b–f
	Rs-RC-69	0.010 ± 0.002 e	2.417 ± 0.375 fgh	0.212 ± 0.017 def	5.417 ± 0.300 d–k
	Rs-RC-76	0.029 ± 0.005 bc	3.583 ± 0.271 b–g	0.237 ± 0.029 b–e	5.750 ± 0.423 c–h
	Rs-RC-82	0.023 ± 0.008 b	3.750 ± 0.382 b–f	0.220 ± 0.015 c–f	5.917 ± 0.271 b–f
	Rs-RC-105	0.013 ± 0.005 cde	3.000 ± 0.258 c–h	0.178 ± 0.033 fgh	4.950 ± 0.263 h–k
AG 4 HG-III	Rs-RC-108	0.025 ± 0.009 bcd	3.833 ± 0.211 a–e	0.218 ± 0.010 c-f	5.667 ± 0.401 c–i
	Rs-RC-109	0.026 ± 0.002 bcd	4.083 ± 0.490 a–d	0.198 ± 0.022 def	5.550 ± 0.293 c–j
AG 5	Rs-RC-114	0.027 ± 0.009 bcd	3.917 ± 0.712 a–e	0.205 ± 0.020 def	5.917 ± 0.300 b–f
	Rs-RC-118	0.018 ± 0.005 cde	3.833 ± 0.833 b–f	0.263 ± 0.023 bcd	5.800 ± 0.163 b–g
	Rs-RC-122	0.018 ± 0.007 cde	3.167 ± 0.558 b–h	0.257 ± 0.021 bcd	6.333 ± 0.247 abc
	Rs-RC-123	0.026 ± 0.013 bcd	3.950 ± 0.419 a–e	0.262 ± 0.013 bcd	6.667 ± 0.401 ab
AG-A	R-RC-125	0.029 ± 0.006 bc	4.000 ± 0.606 a–e	0.297 ± 0.042 bc	6.200 ± 0.100 a–d
	R-RC-127	0.057 ± 0.013 b	4.500 ± 0.548 ab	0.315 ± 0.041 b	6.083 ± 0.239 a–e
	R-RC-129	0.046 ± 0.005 b	4.167 ± 0.792 a–d	0.292 ± 0.026 bc	6.250 ± 0.310 a–d
	R-RC-131	0.027 ± 0.007 bcd	4.250 ± 0.250 abc	0.232 ± 0.031 cde	6.183 ± 0.290 a–d
Control	Control	0.203 ± 0.018 a	5.333 ± 0.558 a	0.558 ± 0.049a	6.917 ± 0.271 a

^a^*Rhizoctonia* spp. isolates obtained from red cabbage. ^b^ Values represent the mean of six replications for each isolate. ^c^ Mean values followed by standard error of the mean. ^d^ Means in a column followed by the same letter are not significantly different according to Fisher’s LSD test (*p* < 0.05).

**Table 4:** Host response of some commercial red cabbage cultivars to isolates belonging to different anastomosis groups (AGs) of *Rhizoctonia* spp. in vivo experiment.

Isolate	Disease Severity Observed on Variety ^a^					
	Rescue	Travero	Integro	Remala	Redriver	Rondale
AG 4 HG-I	4.0 ^b^ ± 0.00 ^c^ b ^d^	4.0 ± 0.00 b	4.0 ± 0.00 b	4.0 ± 0.00 c	4.0 ± 0.00 c	4.0 ± 0.00 c
AG 4 HG-III	4.0 ± 0.00 b B ^e^	4.0 ± 0.00 b B	4.0 ± 0.00 b B	3.3 ± 0.21 bc A	3.5 ± 0.22 bc AB	3.5 ± 0.22 bc AB
AG 5	4.0 ± 0.00 b B	4.0 ± 0.00 b B	4.0 ± 0.00 b B	3.0 ± 0.00 b A	2.5 ± 0.22 b A	4.0 ± 0.00 c B
AG-A	3.0 ± 0.26 a B	3.0 ± 0.00 a B	3.2 ± 0.31 b B	2.7 ± 0.21 b AB	2.3 ± 0.21 ab A	3.2 ± 0.17 ab B
Control	1.0 ± 0.00 a	1.0 ± 0.00 a	1.0 ± 0.00 a	1.0 ± 0.00 a	1.0 ± 0.00 a	1.0 ± 0.00 a

^a^ Root symptoms were evaluated on the following scale: 1 = no symptom; 2 = basal rot on part of stem; 3 = basal rot girdled stem superficially, but plants did not wilt; and 4 = basal rot deeply girdled stem, stem base was constricted, roots were sometimes detached from stem, and plants had wilted. ^b^ Values represent the mean of six replications for each isolate. ^c^ Mean values followed by standard error of the mean. ^d^ Means in a column followed by the same small letters are not significantly different according to Scheirer–Ray–Hare (SRH) test (*p* < 0.05). ^e^ Means in a row followed by the same capital letters are not significantly different according to Scheirer–Ray–Hare (SRH) test (*p* < 0.05).

## Data Availability

All relevant data are within the manuscript.
